# Endothelial Targeted Strategies to Combat Oxidative Stress: Improving Outcomes in Traumatic Brain Injury

**DOI:** 10.3389/fneur.2019.00582

**Published:** 2019-06-06

**Authors:** Evan M. Lutton, S. Katie Farney, Allison M. Andrews, Vladimir V. Shuvaev, Gwo-Yu Chuang, Vladimir R. Muzykantov, Servio H. Ramirez

**Affiliations:** ^1^Department of Pathology and Laboratory Medicine, Lewis Katz School of Medicine at Temple University, Philadelphia, PA, United States; ^2^Vaccine Research Center, National Institute of Allergy and Infectious Diseases, National Institutes of Health, Bethesda, MD, United States; ^3^Center for Substance Abuse Research, Lewis Katz School of Medicine at Temple University, Philadelphia, PA, United States; ^4^Department of Systems Pharmacology and Translational Therapeutics, Perelman School of Medicine, University of Pennsylvania, Philadelphia, PA, United States; ^5^Shriners Hospitals Pediatric Research Center, Lewis Katz School of Medicine at Temple University, Philadelphia, PA, United States

**Keywords:** traumatic brain injury, oxidative stress, catalase, blood-brain barrier, nanomedicine

## Abstract

The endothelium is a thin monolayer of specialized cells that lines the luminal wall of blood vessels and constitutes the critical innermost portion of the physical barrier between the blood and the brain termed the blood-brain barrier (BBB). Aberrant changes in the endothelium occur in many neuropathological states, including those with high morbidity and mortality that lack targeted therapeutic interventions, such as traumatic brain injury (TBI). Utilizing ligands of surface determinants expressed on brain endothelium to target and combat injury mechanisms at damaged endothelium offers a new approach to the study of TBI and new avenues for clinical advancement. Many factors influence the targets that are expressed on endothelium. Therefore, the optimization of binding sites and ideal design features of nanocarriers are controllable factors that permit the engineering of nanotherapeutic agents with applicability that is specific to a known disease state. Following TBI, damaged endothelial cells upregulate cell adhesion molecules, including ICAM-1, and are key sites of reactive oxygen species (ROS) generation, including hydrogen peroxide. Reactive oxygen species along with pro-inflammatory mediators are known to contribute to endothelial damage and loss of BBB integrity. The use of targeted endothelial nanomedicine, with conjugates of the antioxidant enzyme catalase linked to anti-ICAM-1 antibodies, has recently been demonstrated to minimize oxidative stress at the BBB and reduce neuropathological outcomes following TBI. Here, we discuss targeted endothelial nanomedicine and its potential to provide benefits in TBI outcomes and future directions of this approach.

## Introduction to Traumatic Brain Injury

Traumatic brain injury is a growing healthcare concern with 2.5 million TBI-related emergency department visits, hospitalizations, and deaths occurring in the United States in 2013 (1). With increased awareness and widespread media coverage in recent years, the number of reported TBI events continues to climb with TBI being recognized earlier and with increased frequency (2). While the majority of TBI events are mild and present clinically as a concussion, moderate, and severe TBI events have more profound clinical presentations, including but not limited to loss of consciousness, focal neurological deficits, hemorrhage, and cerebral edema, and are associated with higher rates of morbidity and mortality (3). Although technological and clinical advancements in recent decades have improved the quality and length of life for those suffering from TBI, no effective therapy specific for TBI management currently exists in practice. Despite early diagnosis and a therapeutic window for acute intervention, many patients still face long-term morbidity. Several preclinical and clinical studies have been conducted to investigate the benefit of monotherapies and combination therapies in TBI; however, few have been successful in improving patient outcomes (4, 5). Consequently, patients suffering from TBI are faced with limited supportive treatment options and physical rehabilitation, with extensive recovery times and permanent disability.

Few treatment options exist to facilitate functional recovery following TBI (6–8). Current neurorehabilitation efforts for individuals with severe TBI include sensory stimulation and pharmacologic agents, such as amantadine (9, 10). A recent international multicenter randomized clinical trial, POLAR-RCT, demonstrated no benefit of early prophylactic hypothermia in patients with severe TBI (11). This is but one example of the many multicenter trials that have been unable to improve outcomes for TBI patients, especially those with moderate to severe TBI. There is a persistent need in the fields of TBI research and clinical management to continue the search for specific therapeutic strategies that prevent the propagation of secondary injury mechanisms that occur following a traumatic insult to the brain.

## Brain Injury Mechanisms: Inflammation and Oxidative Stress

The pathophysiology of TBI has been described to occur in two sequential, interconnected phases (12). First, primary injury to the brain occurs at the time of impact. Mechanisms of primary injury include contusion to the brain, diffuse axonal injury as neurons are sheared by acceleration and deceleration forces, brain swelling, and intracranial hemorrhage. These injury mechanisms result in focal necrotic cell death and are due to the mechanical insult of TBI (13). Secondary injury mechanisms ensue in response to cell death, release of cellular contents, and diffuse cellular damage. These mechanisms include oxidative damage, BBB disruption, and neuroinflammation. Secondary injury mechanisms overwhelm the body's natural regulatory processes and can lead to exacerbated brain injury (14). Furthermore, these processes begin immediately following the traumatic event and can continue days to weeks after TBI (15). Remarkably, chronic neuroinflammation has been identified up to 16 years after TBI in long-term survivors of head trauma (16). The dynamic pathophysiology and extensive morbidity of TBI, in addition to limited current treatment modalities, demonstrate the dire need for therapeutic interventions targeted against specific mechanisms of secondary injury.

Following injury to the brain, there is an immediate increase in superoxide production, which initiates the cascade of the oxidative stress response (17). Superoxide is rapidly dismutated to hydrogen peroxide and oxygen by the antioxidant enzyme superoxide dismutase (SOD). Hydrogen peroxide, which is known to be more stable than superoxide, is then available to interact with nearby biological substrates, thus propagating oxidative damage in the brain. We have demonstrated rapid free radical production following experimental TBI by controlled cortical impact (CCI-TBI) using dihydroethidium in a novel *in vivo* approach to ROS detection (18). These results coincide with findings that ROS and free radical production are detrimental to TBI outcomes (19). Continuing down the oxidative stress pathway, hydroxyl radical-induced lipid peroxidation is a major mechanism of cellular (membranous) damage in TBI. Intervening with ROS biochemical reactions at the level of hydrogen peroxide has the potential to eliminate subsequent oxidative reactions and cellular injury via this pathway. Congruent with existing literature, increased levels of hydrogen peroxide were found 4 h after CCI-TBI, which may reflect increases seen in downstream oxidative stress pathways such as lipid peroxidation, increased 3-nitrotyrosine levels, and ADP ribosylation as have been demonstrated previously (18, 20–22). The oxidative stress response to TBI has also been shown to affect cerebral blood flow, and thereby directly correlates with cerebral perfusion and oxygenation of brain tissue (23, 24). Unfortunately, the production of ROS following TBI is heterogeneous and unregulated, making it difficult to assess the isolated impact of oxidative stress on cerebral blood flow after injury *in vivo* (25). The sources of oxidative stress following TBI are multiple including cellular and molecular pathways occurring in various cell types such as activated endothelial cells, astrocytes, and microglia, as well as damaged neurons. The cumulative effect of ROS production following traumatic injury to the brain seems to be increased damage to the brain parenchyma with neurons loss, propagated inflammation, and a global increase in cerebral blood flow with concomitant loss of autoregulatory function (24, 26).

While knowledge of the pathophysiological mechanisms that contribute to the morbidity of TBI continues to expand, it is clear that oxidative stress plays a central role in the evolution and progression of traumatic injury to the brain, and inventions targeted to the oxidative stress pathway may improve injury outcomes.

## Animal Models of Traumatic Brain Injury

Over the past few decades, animal models have been developed to replicate various aspects of human TBI and to better understand the underlying pathophysiological mechanisms that propagate injury and to explore potential strategies to prevent long term morbidity associated with TBI. While larger animals such as pigs are closer in size and physiology to humans, rodents are more ubiquitously used in TBI research due to their modest cost and standardized outcome measurements. Four specific models are popularly used in research to recapitulate the effects of TBI in a variety of settings: fluid percussion injury (27), CCI-TBI (28, 29), weight drop injury (30), and blast injury (31). Additionally, novel *in vitro* microfluidic models of the neurovascular unit including dynamic stretch and shear forces as well as computational models of traumatic brain injury are emerging (28, 32). For our purposes, we employed the CCI-TBI model in the mouse for its reproducibility, focal delivery of injury to the brain, and compatibility with the literature utilizing endothelial targeted antibody antioxidant enzyme conjugates.

## Endothelial Targeted Antioxidant Enzyme Therapy

The pharmacologic treatment of TBI is the great challenge, to say the least (33). The endothelial cell layer of the BBB is its luminal most component and regulates the access of blood borne materials into the brain (28). Endothelial cells line the vascular lumen and thereby play a key role in controlling vascular tone, blood fluidity, and extravasation of blood components into tissue parenchyma. Endothelial dysfunction and injury caused by various pathological factors including trauma, inflammatory mediators, and oxidants results in vascular inflammation, oxidative stress, thrombosis, and ischemia, intertwined and mutually propagating processes that are implicated in the pathogenesis of many disease states including TBI. The endothelium represents an important therapeutic target for conditions involving oxidative stress and inflammation in the CNS, such as TBI.

Endothelial targeting through the use of antibodies to cell adhesion molecules is an area of high interest, as it may enable unique pharmacological interventions such as selective targeting of reactive molecules to activated endothelium and trafficking to endothelial organelles, including endosomes, and transcytosis across the endothelial barrier (34). Targeted endothelial nanomedicine agents have been shown to provide antioxidant, anti-inflammatory, and other therapeutic effects that have been unattainable by non-targeted methods in animal models of acute human disease conditions, including TBI (1, 18, 34). The efficacy of targeted therapeutics to the injured endothelium in preclinical models warrants further investigation and offers the potential for translation into the clinical domain. Targeting biotherapeutics to activated endothelium using affinity ligands, such as antibodies to endothelial cell adhesion molecules, has been studied in other models including the experimental treatment of acute oxidative lung injury; however, prior to the work presented in the following section, endothelial targeting particularly of antioxidant enzymes had not been investigated in the context of TBI.

Previous work utilizing anti-PECAM-1/catalase in an animal model of acute lung injury and other forms of acute vascular oxidative stress demonstrated that antibody antioxidant enzyme conjugates could provide protective effects that were unmatched by untargeted and PEGylated antioxidant enzymes alone (4, 35). A study by Shuvaev et al. demonstrated that anti-PECAM-1/catalase specifically protected mice from lung injury induced by hydrogen peroxide produced by glucose oxidase deposited in the pulmonary vasculature. Furthermore, anti-PECAM-1/catalase reduced the extent of alveolar edema and attenuated the decline in arterial oxygen content in mice that underwent unilateral lung ischemia and reperfusion (36).

The first study to use endothelial targeted antioxidant enzymes to attenuate damage in the central nervous system utilized two models of endothelial insult in the brain. First, an experimental model of cerebral inflammation was performed in mice following intraperitoneal injection of LPS to model sepsis and cerebrovascular encephalitis. Exposure to LPS resulted in inflammatory upregulation of cell adhesion molecule VCAM-1 expressed on cerebral vascular endothelial cells. Second, the middle cerebral artery occlusion model of stroke was employed to demonstrate the utility of endothelial targeted antioxidant enzyme therapy in reducing infarct volume after ischemic insult to the brain. In the model of endotoxin encephalitis, LPS administration caused firm adhesion of leukocytes to the cerebral vasculature, assessed by intravital microscopy via implanted cranial window. Endothelial targeted antioxidant enzyme therapy administered concomitantly with LPS attenuated the endotoxin-induced leukocyte adhesion and increased the proportion of cells rolling along the endothelium. The agent administered in these studies was a conjugate of SOD to anti-PECAM-1 antibodies. These findings demonstrated that anti-PECAM-1/SOD provided anti-inflammatory effects *in vivo* which was consistent with previous findings in *in vitro* models (33, 34, 37, 38).

Other studies have assessed the utility of various strategies to improve the delivery of antioxidant enzymes including PEGylation, the use of fusion constructs with protein transduction domains, and encapsulation of antioxidant enzymes in liposomes (39–41). Such approaches are limited by the stability of these compounds in circulation, delivery potential to and across the blood-brain barrier, and concerns regarding systemic toxicity. Furthermore, since ROS act on very short distances, on the scale of angstrom to a few nanometers, very precise delivery of antioxidants to the site of ROS production is necessary. Additional research strategies to combat secondary mechanisms of injury following TBI have utilized other non-targeted nanotherapeutic approaches including systemic administration of exosomes derived from mesenchymal stem cells (42).

Importantly, the premise of endothelial targeted intervention strategies for TBI studies relies on changes to the endothelium following insult and activation. Endothelial activation, an early event following TBI, has been detailed by demonstrating increased expression of cell adhesion molecules, including ICAM-1, using the CCI-TBI model. We demonstrated marked increases in ICAM-1 expression on cortical vasculature up to 48 h following CCI-TBI, highlighting ICAM-1 as an appropriate target for the delivery of nanotherapeutics to activated endothelium in the site of injury at acute time points. Upregulation of ICAM-1 and other adhesion molecules by the cerebral endothelium following TBI is well established, and our results support previous findings of early endothelial activation following TBI, including *in vitro* studies and recent work with the CCI-TBI model (43–45).

## Beneficial Effects of Anti-ICAM-1/Catalase in an Experimental Model of TBI

**Figure 1:**
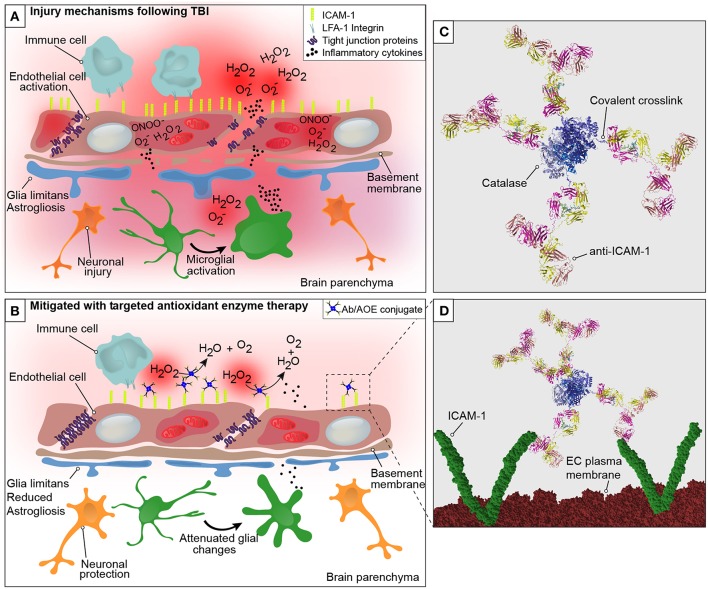
Graphical representation of the neurovascular unit and blood-brain barrier following traumatic brain injury with theoretical mechanism of action of anti-ICAM-1/catalase. The blood-brain barrier is comprised of endothelial cells that express tight junction proteins, which restrict the paracellular transfer of solutes and proteins between and blood and brain. The glia limitans, or astrocytic end feet, also contribute to the physical properties of the barrier. This establishes a delicate homeostasis in the brain that is required for normal glial and neuronal functioning. The above figure is a simplified representation of the aspects of the BBB that have been investigated in the context of endothelial targeted antioxidant enzyme therapy for TBI and is not meant to serve as a complete illustration of BBB structure or biology. **(A)** Following disruption to the neurovascular unit and blood-brain barrier, as occurs in TBI, secondary mechanisms of injury including oxidative stress, endothelial activation, central nervous system, and peripheral inflammation take place that propagate lesion size and increase damage in the brain. The production of reactive oxygen species by endothelial cells is one of the earliest events to occur with endothelial damage and neurotrauma, depicted here are superoxide anion (O${}_{2}^{-}$), hydrogen peroxide (H_2_O_2_), and peroxynitrite (ONOO^−^). With endothelial activation, endothelial cells upregulate the surface expression of ICAM-1, and tight junction complex disassembly leads to hyperpermeability of the blood-brain barrier. Continued oxidative stress processes result from cell death and activation of microglia and astrocytes, which produce inflammatory mediators and cytokines to recruit peripheral immune cells to the site of injury. **(B)** Early intervention of oxidative stress processes with the administration of anti-ICAM-1/catalase acutely following CCI-TBI provided neuroprotective, glial protective, and BBB protective effects after injury. Targeted delivery of catalase, an antioxidant enzyme that converts hydrogen peroxide (H_2_O_2_) to water and oxygen, to ICAM-1 preserved BBB structure and function, limited glial activation, and offered neuroprotection with increased neuronal detection in the cortical area of TBI. **(C)** Ribbon structure representation of anti-ICAM-1/catalase conjugate. Antibodies to ICAM-1 are depicted with covalent linkage to tetrameric recombinant catalase. **(D)** Following acute administration of anti-ICAM-1/catalase to mice after experimental TBI, we hypothesize that anti-ICAM-1/catalase is targeted based on antibody specificity to ICAM-1, which is upregulated on the activated endothelial cells of the BBB. Depicted here in the ribbon structure representation of the anti-ICAM-1/catalase conjugate interacting with dimeric ICAM-1 molecules on the luminal endothelial cell (EC) plasma membrane.

In a recent study, we demonstrated the use of anti-ICAM-1/catalase as an acute intervention to combat oxidative stress following experimental TBI (18). We investigated whether the targeted delivery of anti-ICAM-1/catalase in the context of TBI would interfere with the oxidative stress response to traumatic injury and thus preserve BBB integrity, alleviating the neuropathological findings associated with TBI. To our knowledge, this is the first assessment of an endothelial targeted enzymatic approach to preventing oxidative stress as a secondary injury mechanism in TBI. Traumatic brain injury was shown to cause a dramatic increase to ROS production and endothelial activation in the brain compared to uninjured shams and naive animal controls. This is consistent with previous reports in the literature and offers an early target for intervention of secondary injury mechanisms following TBI. Acute administration of anti-ICAM-1/catalase at 30 min following CCI-TBI resulted in decreased cerebral hydrogen peroxide production, decreased markers of protein nitrosylation, preserved or protected BBB integrity, and reduced indices of neuropathological markers specific for inflammatory changes after brain injury (graphical representation in Figure 1) (46). See Video S1 for three-dimensional visualization of anti-ICAM-1/catalase (47).

Early intervention of oxidative stress processes with anti-ICAM-1/catalase administered acutely following CCI-TBI provided neuroprotective, glial protective, and BBB protective effects after injury. The production of ROS by endothelial cells is one of the earliest events to occur with endothelial damage and neurotrauma. With endothelial activation, endothelial cells upregulate the surface expression of ICAM-1, and tight junction complex disassembly contributes to hyperpermeability of the BBB. In addition to the effects on tight junction proteins, ROS have been shown to dysregulate transporter proteins and increase caveolae-mediated transcytosis (48, 49). Targeted delivery of catalase, an antioxidant enzyme that converts hydrogen peroxide to water and oxygen, to ICAM-1 preserved BBB structure and function, limited glial activation, and offered neuroprotection with increased neuronal detection in the cortical area of injury in TBI.

**Figure 2:**
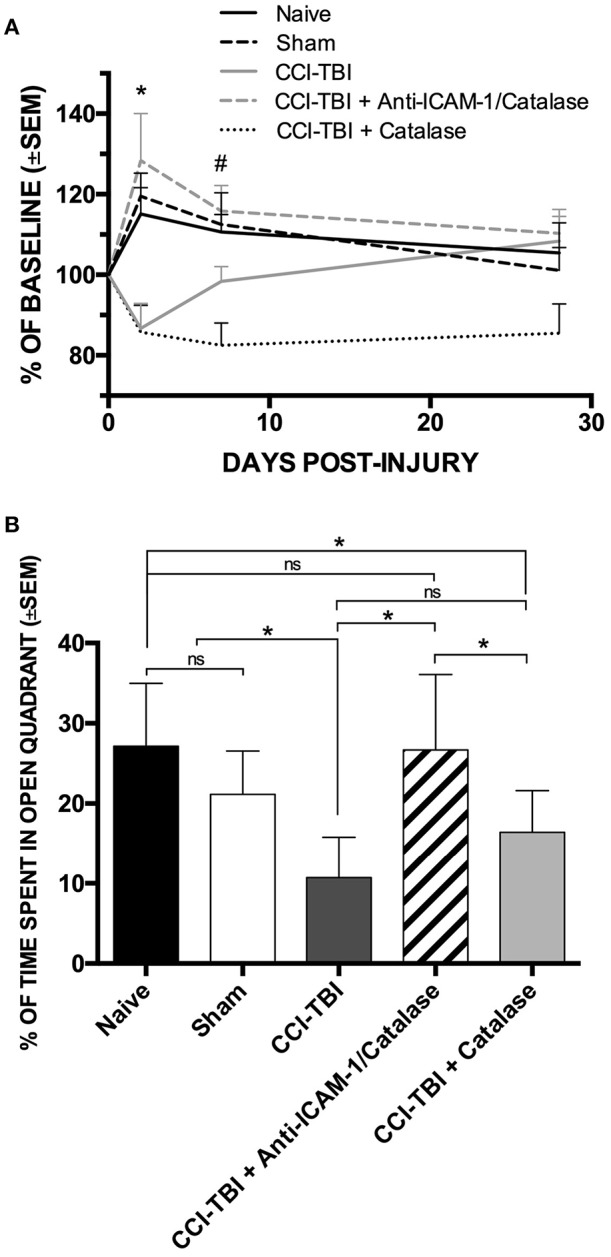
Anti-ICAM-1/catalase improves acute motor performance and normalizes anxiety phenotype in behavioral assessment following experimental TBI. **(A)** Rotarod testing was performed at 48 h, 1 week, or 4 weeks after injury for naive, sham, and CCI-TBI mice as well as mice receiving either anti-ICAM-1/catalase or catalase alone at 30 min post-CCI-TBI. At 48 h, CCI-TBI + Anti-ICAM-1/Catalase group and sham group significantly outperformed CCI-TBI and CCI-TBI + Catalase groups. At 1 week post-CCI-TBI significantly greater performance was maintained by the CCI-TBI + Anti-ICAM-1/Catalase group over the mice receiving a CCI-TBI followed by catalase alone. Significantly differences were lost by 4 weeks post-CCI-TBI. Data presented as mean latency to fall per group as a percentage of individual baseline performance (mean ± SEM) (ordinary one-way ANOVA. At 48 h ^*^F = 6.301, *P* = 0.0007. At 1 week #*F* = 5.736, *P* = 0.0012). **(B)** Elevated zero maze testing was performed at 4 weeks following CCI-TBI for naive, sham, and CCI-TBI mice as well as mice receiving either anti-ICAM-1/catalase or catalase alone at 30 min post-injury. No significant difference was found between naive or sham animals for percentage of time spent in the open quadrants of the maze. With CCI-TBI, animals spent significantly less time in the open quadrants indicating an increased anxiety-like phenotype. Anti-ICAM-1/catalase gave significantly greater time spent in the open quadrants than CCI-TBI and CCI-TBI treated with catalase alone. There was no significant difference between CCI-TBI and CCI-TBI + Catalase groups. Data are presented as mean time spent in open quadrants as a percentage of total trial time (mean ± SEM) (ordinary one-way ANOVA with Dunnett's *post-hoc* test. *F* = 8.145, *P* = 0.0001).

The protective effects seen on the biochemical and cellular level offers potential for functional improvement with acute anti-ICAM-1/catalase administration following TBI. To assess functional outcomes after TBI, we performed a battery of behavioral analyses to investigate changes in motor, anxiety, and cognition after TBI with and without treatment using anti-ICAM-1/catalase. Detailed methods for all behavioral assays are provided in the Supplemental Materials. The Rotarod assay for motor and coordination function was performed at baseline and at 48 h, 1 week, and 4 weeks following CCI-TBI. Passive and active latency were recorded for all trials with the passive latency endpoint defined as the time at which the mouse underwent one full rotation without fall and active latency defined as the time at which the mouse fell from the Rotarod. At 48 h following CCI-TBI, the CCI-TBI + Anti-ICAM-1/catalase group and sham group significantly outperformed CCI-TBI and CCI-TBI + non-targeted catalase groups with greater time until active latency as determined by percentage of baseline performance (Figure 2A). At 1 week post-CCI-TBI significantly greater performance was maintained by the CCI-TBI + anti-ICAM-1/catalase group over the mice receiving a CCI-TBI followed by catalase alone. However, other differences were lost as the groups uniformly converged toward baseline performance over time. These results suggest an improved motor benefit with administration of anti-ICAM-1/catalase, especially in the acute time period after injury.

Traumatic brain injury is known to increase the incidence of anxiety disorders in human patients (50). To assess anxiety-like phenotypes in mice receiving CCI-TBI, the elevated zero maze was used at 4 weeks post-CCI-TBI, as previously described (51). No significant difference was found between naive or sham animals for percentage of time spent in the open quadrants of the maze, indicating that the process of the sham surgery did not increase anxiety-like behavior at the 4-weeks time point. Following CCI-TBI, animals spent significantly less time in the open quadrants indicating an increased anxiety-like phenotype compared to naive and sham animals. Anti-ICAM-1/catalase normalized anxiety-like behavior with animals of this group spending significantly greater time spent in the open quadrants than the CCI-TBI groups. CCI-TBI treated with recombinant catalase alone did not significantly improve performance in the elevated zero maze above that seen in the untreated CCI-TBI group, supporting the benefit of targeted antioxidant enzyme therapy (Figure 2B).

Traumatic brain injury can also impede cognitive performance in the acute phase as well as long-term cognition and ability (52, 53). The Barnes maze was utilized to assess cognitive performance in mice in the chronic phase following CCI-TBI. Barnes maze testing was performed at 5 weeks following CCI-TBI for naive, sham, and CCI-TBI mice as well as mice receiving either anti-ICAM-1/catalase or catalase alone at 30 min post-injury. No statistically significant difference was found between groups for improved cognitive performance on the test over time regarding parameters of average latency to the target hole or average percent time spent in the quadrant of the target hole (Figure S1). While no significant differences were found, sham and anti-ICAM-1/catalase groups trended toward improved cognitive function compared to CCI-TBI and CCI-TBI + untargeted recombinant catalase. Taken together, these additional findings further augment the convincing proof-of-concept data that targeted antioxidant enzyme therapy as an effective potential treatment strategy for early intervention after TBI.

## Conclusions and Future Directions

Traumatic brain injury continues to be a major clinical and socioeconomic healthcare concern with very limited treatment options specific to its clinical and pathophysiological course. Innovative research in the field of targeted nanotherapeutics has permitted the delivery of enzymatic compounds to the BBB, where they can act against specific aspects of secondary injury propagation after TBI, like the oxidative stress response. We have shown improved histopathological and behavioral outcomes in an experimental model of TBI with administration of catalase targeted to ICAM-1. These findings support the continued research of antibody antioxidant enzyme conjugate therapy as a means to intervene early after traumatic injury to the brain.

Current work in antibody targeted antioxidant enzyme therapy aims to expand our findings with the use of anti-ICAM-1/catalase in experimental TBI to optimize dosing strategies and the therapeutic window for intervention. We also expand upon our arsenal of therapeutic carriers including liposomes and polymeric nanocarriers (54, 55), alternative or additional antioxidants such as SOD together with catalase, and anti-inflammatory, and anti-thrombotic agents (56–59). Recent studies indicate that precise targeting to specific endothelial endosomes markedly enhances protective effect of antioxidant enzymes (60, 61). Such precision requires controlled configuration of the conjugates, their size, amino acid modification, and packaging into nanocarriers (62–66). Furthermore, replacement of whole antibody molecules by scFv fragments has been shown to further improve therapeutic outcomes (67).

Many exciting models of TBI are being developed that are amenable to the study of nanotherapeutic intervention of oxidative stress and inflammatory processes. However, to date these models do not recapitulate the architecture of the neurovascular unit, blood flow and all contributing cell types in one model. Andrews and colleagues utilized an *in vitro* mechanical strain injury model to stretch human brain endothelial cells and study the endothelial production of extracellular microvesicles following simulated TBI (28). Extracellular microvesicles are known to be key regulators of intercellular communication, however their role in the pathogenesis of TBI is not clearly understood. Furthermore, Andrews et al. demonstrated that the microvesicles contained endothelial markers including the tight junction protein occludin (28). It remains unknown the role of extracellular microvesicles in the cascade of oxidative stress and neuroinflammation following TBI or how these processes may influence microvesicle production and BBB breakdown via this pathway. Mechanical strain insult has been previously applied to neurons to demonstrate axonal beading and microtubule breakdown following simulated TBI (68). Other *in vitro* models of TBI utilize stretch injury along with oxygen and glucose deprivation with different cellular compositions and “brain on a chip” platforms (69, 70). Incorporations of the strengths of these models should be considered which could result in productive collaborations to better understand and target pathophysiological processes of TBI to discover novel therapeutic advances.

New targeted pharmacological agents and novel strategies of modeling TBI will help to assess neuropathological and neurological outcomes of injury with interventions timed to interfere with different aspects of the oxidative, inflammatory, and thrombotic stress responses. Additionally, novel *in vitro* models may be employed to continue the study of antibody antioxidant enzyme complexes in a system that recapitulates multiple components of the neurovascular unit and permits the study of the effects of fluid flow, injury, and intervention on various cell types that contribute to the pathogenesis of TBI. Overall, this work and these findings will contribute to the advancement of research regarding therapeutic strategies to establish early interventions against secondary injury mechanisms, a prominent obstacle in the clinical advancement and translation of therapies for TBI and many other neuropathological conditions.

## Institutional Assurances

Temple University's Institutional Animal Care and Use Committee (IACUC) approved all procedures detailed under Figure 2 requiring the use of vertebrate animals prior to initiating any experimental objectives. The methods and techniques used were in full compliance with Temple University's IACUC policies and the National Institutes of Health (NIH) ethical guidelines. Animal housing and procedures were performed at Temple University's Central Animal Facility.

## Ethics Statement

The Institutional Animal Care and Use Committee (IACUC) at Temple University (Philadelphia, PA) approved all procedures detailed in studies presented herein that required the use of vertebrate animals prior to initiating any experimental objectives. Additionally, all methods were performed in full compliance with Temple University's IACUC policies and the National Institutes of Health (NIH) ethical guidelines. Animals were housed and allowed to acclimate for 1–2 weeks in the Temple University Central Animal Facility. The animals were provided standard environmental enrichment conditions and were fed with a commercial pellet diet and water *ad libitum*. Eight-week-old male C57BL/6J were obtained from The Jackson Laboratory (Bar Harbor, ME).

## Author Contributions

All authors listed have made a substantial, direct and intellectual contribution to the work, and approved it for publication.

### Conflict of Interest Statement

The authors declare that the research was conducted in the absence of any commercial or financial relationships that could be construed as a potential conflict of interest.

## Supplementary Material

Supplementary Figure 1 Anti-ICAM-1/catalase trends toward improved cognitive function as evaluated by the Barnes maze. Barnes maze testing was performed at 5 weeks following CCI-TBI for naive, sham, and CCI-TBI mice as well as mice receiving either anti-ICAM-1/catalase or catalase alone at 30 min post-injury. No statistically significant difference was found between groups for improved cognitive performance on the test over time regarding parameters of **(A)** average latency to the target hole or **(B)** average percent time spent in the quadrant of the target hole. While no significant differences were found, sham and anti-ICAM-1/catalase groups trended toward improved cognitive function compared to CCI-TBI and CCI-TBI + catalase (ordinary one-way ANOVA. *F* = 6.368, *P* = 0.0236).

Supplementary Video 1 Three-dimensional video representation of anti-ICAM-1/catalase. Ribbon structure representation of anti-ICAM-1/catalase conjugate. Antibodies to ICAM-1 are depicted with covalent linkage to tetrameric recombinant catalase. Molecular graphics and analyses performed with UCSF Chimera, developed by the Resource for Biocomputing, Visualization, and Informatics at the University of California, San Francisco.

Data Sheet 1 Supplemental Methods.
