# Supplementary material for: Endothelial Targeted Strategies to Combat Oxidative Stress: Improving Outcomes in Traumatic Brain Injury
Source: Front Neurol. 2019 Jun 6;10:582. doi: 10.3389/fneur.2019.00582 (PMC6593265; doi:10.3389/fneur.2019.00582)
Supplement: Supplementary Figure 1 — Anti-ICAM-1/catalase trends toward improved cognitive function as evaluated by the Barnes maze. Barnes maze testing was performed at 5 weeks following CCI-TBI for naive, sham, and CCI-TBI mice as well as mice receiving either anti-ICAM-1/catalase or catalase alone at 30 min post-injury. No statistically significant difference was found between groups for improved cognitive performance on the test over time regarding parameters of (A) average latency to the target hole or (B) average percent time spent in the quadrant of the target hole. While no significant differences were found, sham and anti-ICAM-1/catalase groups trended toward improved cognitive function compared to CCI-TBI and CCI-TBI + catalase (ordinary one-way ANOVA. F = 6.368, P = 0.0236). [file Image_1.pdf]

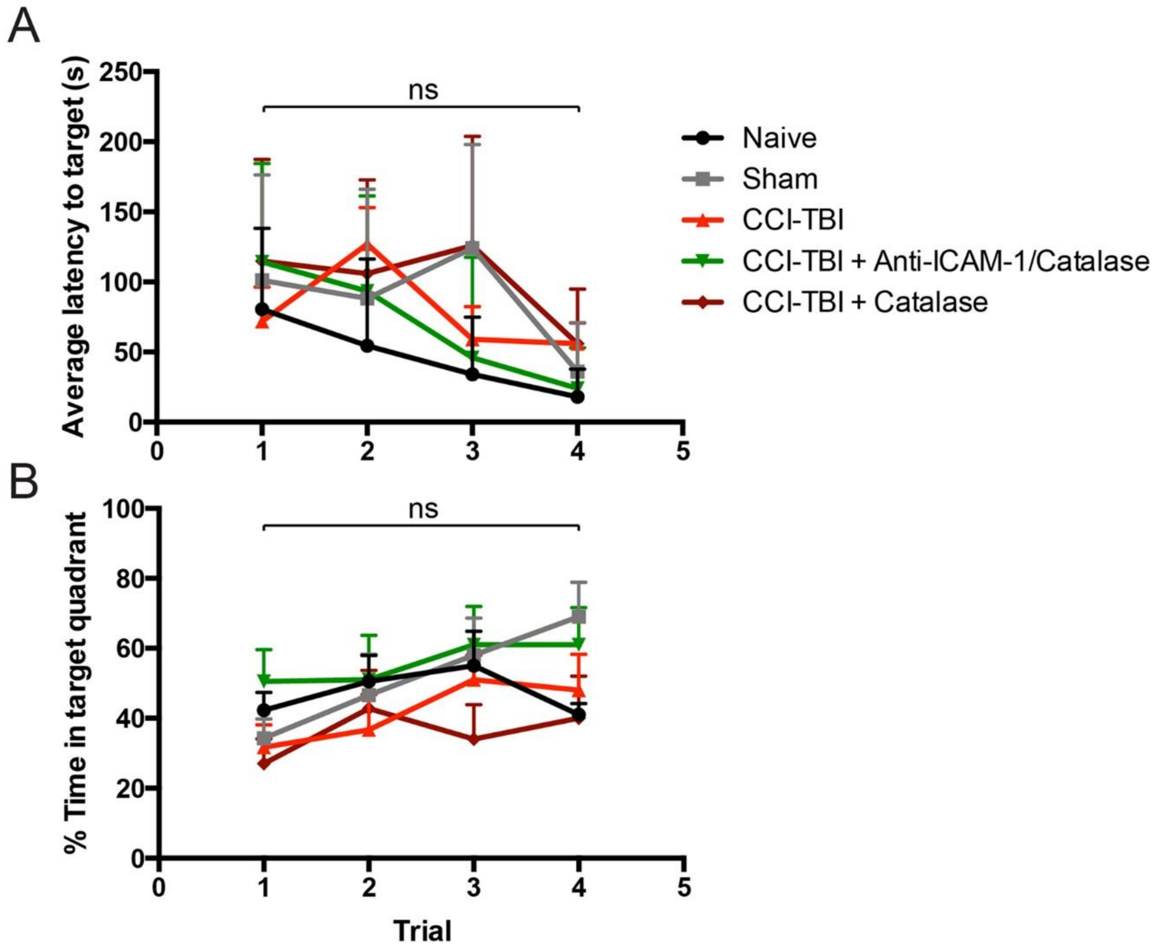


**Figure S1. Anti-ICAM-1/catalase trends toward improved cognitive function as evaluated by the Barnes maze.**
