# Supplementary material for: Endothelial Targeted Strategies to Combat Oxidative Stress: Improving Outcomes in Traumatic Brain Injury
Source: Front Neurol. 2019 Jun 6;10:582. doi: 10.3389/fneur.2019.00582 (PMC6593265; doi:10.3389/fneur.2019.00582)
Supplement: Data Sheet 1 — Supplemental Methods. [file Data_Sheet_1.pdf]

Supplemental Methods:

Animals
Behavioral assays performed for the completion of this work included Rotarod (Ugo Basile, Stoelting) for the assessment of motor function and coordination, elevated zero maze (Maze Engineers) for anxiety-like behavior, and Barnes maze (Maze Engineers) for spatial reference memory and learning. Experimental groups included naive (non-surgical controls), sham-operated controls (craniectomy without CCI-TBI), CCI-TBI, CCI-TBI + anti-ICAM-1/catalase, and CCI-TBI + catalase (n=8/group).

The Institutional Animal Care and Use Committee (IACUC) at Temple University (Philadelphia, PA) approved all procedures detailed in studies presented herein that required the use of vertebrate animals prior to initiating any experimental objectives. Additionally, all methods were performed in full compliance with Temple University's IACUC policies and the National Institutes of Health (NIH) ethical guidelines. Animals were housed and allowed to acclimate for 1-2 weeks in the Temple University Central Animal Facility. The animals were provided standard environmental enrichment conditions and were fed with a commercial pellet diet and water ad libitum. Eight-week-old male C57BL/6J were obtained from The Jackson Laboratory (Bar Harbor, ME).

Rotarod Assay
Rotarod training and baseline performance were measured for 40 mice at 6wks of age prior to any surgical procedure. Training was performed for two 180s intervals intervals with a fixed Rotarod speed of 4rpm. Baseline performance was measured on the same day over three 300s trials with accelerating Rotarod speed of 4-40rpm. Mice were given a rest period of 5min between all back-to-back trials. Rotarod performance was then assessed at 48hrs, 1 week, and 4 weeks following CCI-TBI. Passive and active latency was recorded for all trials with passive latency defined as the mouse undergoing one full rotation without fall and active latency defined as the time at which the mouse fell from the Rotarod. Data presented as mean latency to fall per group as a percentage of individual baseline performance (mean±SEM).

Elevated Zero Maze
The elevated zero maze was utilized to assess anxiety-like behavior at 4 weeks following CCI-TBI. Testing was performed with one 5min trial on each of three consecutive days. Mice were placed in a dark quadrant of the maze, and time spent in dark and light quadrants as well as number of entries into light quadrants was recorded using ANY-maze software (Wood Dale, IL). Notably, in the catalase only group, n=3 mice consistently fell from the open quadrants during testing. When this occurred, the mice were replaced in the dark quadrant to restart the trial. Data are presented as mean time spent in open quadrants as a percentage of total trial time (mean±SEM).

Barnes Maze
The Barnes maze was used to study spatial reference memory and learning at 5 weeks following CCI-TBI. Testing was performed in three phases: habituation (day 1), training (day 1-3), and probe trial (day 4). Habituation was performed by placing each mouse in

the center of the maze while the experimenter gently guided the mouse to the escape hole. The escape hole remained in the same location for each phase and trial. Training consisted of 3 3min trials per mouse per day over 3 days. Each trial began when the mouse was placed in the center of the maze. The mouse was allowed to explore the maze for 3min. If at the end of each trial the mouse had not entered the escape hole, the experimenter gently guided the mouse to the hole and allowed it to remain there for 1min. The probe phase consisted of a single 90s trial performed 24hrs following the final training phase. The escape cage was removed, and ANY-maze software was used to track the mice and measured number of attempts made at the escape hole and time spent in the escape hole quadrant. During training phases, ANY-maze software was used to measure latency to target, distance traveled, average motor speed, path efficiency, number of errors, and time spent in the escape hole quadrant. Data are presented as mean latency to escape hole (mean±SEM).

Statistical Analysis
Data were analyzed for statistical significance using Prism software (version 6 GraphPad Software Inc.; La Jolla, CA). Student's t-tests and one-way analysis of variance (ANOVA) with Dunnett's post-hoc tests for multiple comparisons were performed to analyze the biochemical assays and ICAM-1 time course staining. IHC, two-photon imaging, and behavioral data were analyzed by one-way ANOVA followed by Tukey's post-hoc tests. For all tests, statistical significance was defined at $p<0.05$.
